# Professional Integrity

**Published:** 1887-01-01

**Authors:** 


### PROFESSIONAL INTEGRITY.

The object of our journal is to maintain and encourage the honor, the dignity the rights, and the interest of the Profession, and to give its members the cream of medical and scientific discoveries in a brief and concise manner, so that they may be utilized in every-day practice. We have conducted the RECORD successfully for sixteen years, and have had a harmonious career with the masses of the profession, who have ever been with us, and, with very few exceptions, we have been in full accord with our cotemporaries in journalism. We have never differed with any of them, only when we thought that justice to our mutual calling required it to be done, and hoped that good would be accomplished.

In antagonizing wrong, we have incurred the displeasure, and even the enmity, of some who either neglected, or did not know how, to properly estimate the importance and worth of true character, and by authority used by self-constituted leaders stigmatized us as malcontents ; but we have the satisfaction of knowing that we have never, knowingly, violated principle or been untrue to its precepts. We oppose error rather than men, and endeavor to do so with fraternal feelings, and in a manner that will convince the perpetrators of wrong, and also show where it will bring injury to them and to others

True friendship never lays "flattering unction" to a man's soul when he is known to be in error, or seeks to ingratiate itself by pandering to error. The man who is your true friend will come to you with manliness, kindness, and sincerity, and frankly tell you of any wrong you are committing or cherishing, and warn you of the inevitable results, as surely as he will snatch you from a precipice over which you are about to step, blindfolded. If you are capable of appreciating this act of real friendship, instead of taking offence, you will receive it in a grateful manner Reason will tell you your friend is right, and you will confess your error and forthwith endeavor to repair any injury it may have done This spirit of truth and sincerity in friendship is necessary, not only to insure the dignity, harmony and good feeling in all professions and organizations, but is really indispensable to the maintenance of human brotherhood and the progress of an enlightened and Christain civilization

There are men in every State and community who, in their motives and actions, are so largely governed by a spirit of selfishness that they are ready to oppose every man whose deeds are well known to be prompted by established principles of right and impartial justice. Instead of laying themselves open to reasonable conviction, and acknowledge their error, they adopt questionable schemes and plans to set themselves right before the public and their followers. They endeavor to carry their point by proving that right is wrong, and wrong is right, and thus do injury to the good cause they assail, lose their self-respect, and bring shame to themselves and all who have been tricked or led into their methods and measures. It is a sad commentary upon human nature that, sometimes, through selfishness or unreasonable jealousy, professed friends will condemn good men more for doing right than they would for doing wrong; thus verifying the words of Dr. Channing, where he says that "no human being, man or woman, can act up to a sublime standard without giving offense."

The world has always had its martyrs to Truth and the Right, and these heroic spirits will be found so long as darkness is opposed to light, and evil to good. In this age, Truth, instead of maintaining a quiesant defence, is compelled to become more and more aggressive, and the man who remains silent and covers up an error that opposes a good work or does injustice to a worthy individual, is considered accessory to the motive and the deed, and virtually becomes an endorser of the wrong which is contemplated or has been committed.

An old lady, in Hancock County, Ga., once said that "human nature was a grand rascal." The evil that is inherent in all hearts is continually trying to lower the standard of humanity, and even drag it into the mire. To oppose this tendency in ourselves, and in our fellows, and to inculcate truth and integrity of character is the duty of every good man—especially of every instructor of the young, who should be taught to abhor wrong of every kind, and at any cost to stand squarely by the right. These principles, sublime in their character, and great and important in their action, should be taught not only in our homes and churches but in our medical colleges and all institutions of learning.

Of all professions, that of Medicine should have its escutcheon stainless. In its principles, its methods and measures, and in every medical organization the profession should keep its honor intact, and at all times present to its followers and the public a fearless and immovable attitude in defence of Truth and Right. The ethics is plain in all its requirements, and gives to every member of the profession equal rights in every State. It gives emphatic rules in regard to professional honor, courtesy and fraternity alike to its members in every State and community. It allows no member in any locality to be tacitly or openly exempted from the imperative obligations of the professional code. Neither should any member or Society be allowed more privileges under the rules than another, nor should one be exalted to positions of honor and trust who has not honored himself and the fraternity by his life, in word and in deed.

Amid the corruption of political and municipal methods, the public has become accustomed, if not resigned, to seeing the office honor the man rather than the man the office; but in the profession of Medicine—Divine in its origin and Christ-like in its functions—the man should honor the position that he has been appointed to fill. This no unprincipled, selfish trickster can do, whether in political circles or in the pale of the Medical profession. P.

